# Serine Protease MP2 Activates Prophenoloxidase in the Melanization Immune Response of *Drosophila melanogaster*

**DOI:** 10.1371/journal.pone.0079533

**Published:** 2013-11-15

**Authors:** Chunju An, Mingming Zhang, Yuan Chu, Zhangwu Zhao

**Affiliations:** Department of Entomology, College of Agriculture and Biotechnology, China Agricultural University, Beijing, China; Ecole Normale Supérieur de Lyon, France

## Abstract

In arthropods, melanization plays a major role in the innate immune response to encapsulate and kill the invasive organisms. It is mediated by a serine protease cascade and is regulated by serpins. The identification of the molecular components of melanization and the regulation of those components are still unclear in *Drosophila melanogaster*, although some genetic research on the activation of melanization has been reported. Here we report that *Drosophila* serine protease MP2 directly cleaves both recombinant and native prophenoloxidase-1. Overexpression or repression of MP2 in flies resulted in increased and decreased rates of cleavage, respectively, of prophenoloxidase-1. Moreover, serine protease inhibitor Spn27A formed SDS-stable complexes with MP2, both *in vitro* and *in vivo*. The amidase activity of MP2 was inhibited efficiently by Spn27A. Spn27A also prevented MP2 from cleaving prophenoloxidase-1. Taken together, these results indicate that under our experimental conditions MP2 functions as a prophenoloxidase-activating protease, and that this function is inhibited by Spn27A. MP2 and Spn27A thus constitute a regulatory unit in the prophenoloxidase activation cascade in *Drosophila*. The combination of genetic, molecular genetic and biochemical approaches should allow further advances in our understanding of the prophenoloxidase-activating cascade in insects and indirectly shed further light on protease-cascades in humans and other vertebrates.

## Introduction

Insects rely on their innate immune system to defend against invasion by pathogens or parasites [1]. Genetic and molecular approaches have revealed striking similarities between the mechanisms that regulate insect host defense and the mammalian innate immune response. In insect innate immune response, besides the well-documented induction of antimicrobial peptides through the Toll and Imd pathways [2]–[4], melanization functions to encapsulate and kill invading microbes, and also cooperates with other immune responses such as blood coagulation, wound healing, phagocytosis, and antimicrobial peptide expression [5], [6]. Fruit fly, *Drosophila melanogaster*, has extensively been used to study molecular mechanisms involved in the activation and regulation of innate immune responses. However, our knowledge about the melanization response in *Drosophila* is rather limited compared to the characterization of the Toll signaling pathway. Studies on the mechanism of melanization have focused more on relatively large insects such as the silkworm, *Bombyx mori* [7]–[9], the tobacco hornworm, *Manduca sexta* [10]–[12], and the beetle *Tenebrio molitor* [13], [14], none of which have the wealth of genetic techniques that are available in *Drosophila*.

During melanization, phenoloxidase (PO) catalyzes hydroxylation of monophenols to *o*-diphenols and the oxidation of *o*-diphenols to quinones, which then polymerize to form melanin [15]. Melanin and cytotoxic molecules produced in this process, including quinones and reactive oxygen intermediates, may sequester and kill the intruding microorganisms [16]. Arthropod POs are produced as inactive zymogens called prophenoloxidase (PPO), and the activation of PPO is one of the rate-limiting steps in melanization [5]. PPO activation is mediated by a serine protease cascade, which is somewhat analogous to the coagulation pathway and complement system in human plasma [17], [18]. Biochemical studies in *M. sexta* and *T. molitor* have led to the current model of PPO activation [10], [11]–[14], [19]. Soluble pattern-recognition proteins initially recognize non-self molecular patterns in invading organisms or from aberrant host tissues. This interaction triggers activation of a series of serine proteases, culminating in the activation of prophenoloxidase-activating protease (PAP), also known as PPO-activating enzyme or factor (PPAE or PPAF) [10], [11]–[14]. Activated PAP converts inactive PPO to PO by cleaving PPO at an Arg-Phe bond at approximately residue 50 [12], [18], [20]–[24]. PAPs have been identified from several arthropods, including *B. mori* [7], *M. sexta* [12], [18], [20], the mosquito, *Anopheles gambiae* [25], two beetles, *Holotrichia diomphalia* and *T. molitor* [14], [22], and a crayfish, *Pacifastacus leniusculus* [23]. All of these PAPs belong to a family of serine proteases containing a clip domain (a family therefore called clip-domain serine proteases). The proteases have a C-terminal catalytic domain and one or two N-terminal clip domains, connected by a disulfide bond [26]. Genetic evidence in *Drosophila* indicates that three clip-domain proteases MP1 (CG1102), MP2 (CG3066), and Hayan (CG6361) are involved in melanization [27], [28], [29]. Hayan directly converts PPO to PO in systemic wound response [29]. Whether MP1 or MP2 directly activates *Drosophila* PPO is unknown.

PPO activation is highly regulated, presumably because cytotoxic intermediates generated by uncontrolled PPO activation would be harmful to the insect [30]. Serine protease cascades that contribute to PPO activation are often controlled by members of the serine protease inhibitor (serpin) superfamily [31], [32]. Serpins contain ∼400 amino acid residues with an exposed reactive-center loop near their carboxyl terminus [33]. They function as suicide-substrate inhibitors by forming irreversible complexes with target proteases after the cleavage of a scissile bond (designated P1–P1’) in the reactive-center loop [33]–[35]. A group of orthologous serpins which negatively regulate PAPs has been identified in several insects, including *An. gambiae* SRPN2 [25], *M. sexta* serpin-3 [34], *T. molitor* Spn48 [35]. Six serpins, including Spn27A, Spn28D, Spn43Ac, Spn77Ba, Spn4, and Spn5, have been studied functionally in *Drosophila* [36]–[42]. Spn27A inhibits Easter in the establishment of embryonic dorsoventral polarity, and also controls an unknown protease in melanization [36], [43], [44]. Recombinant Spn27A inhibits *H. diomphalia* PPAE *in vitro* [36], but has no direct regulation on Hayan [29]. The endogenous targets of Spn27A in *Drosophila* are still unknown.

Our current knowledge about melanization in *Drosophila* is mainly obtained from genetic studies. Here we report methods to activate purified recombinant MP2. We then use this purified enzyme in biochemical assays - in combination with genetic techniques - to investigate the molecular mechanisms of *Drosophila* PPO activation. Our results indicate that MP2 directly cleaves and activates *Drosophila* prophenoloxidase 1 (PPO1) and, therefore, functions as a PAP in *Drosophila*. Moreover, Spn27A is found to inhibit MP2 activity. MP2 and Spn27A thus constitute a regulatory unit in the PPO activation cascade in *Drosophila*.

## Results

### Phylogenetic Analysis of *Drosophila* Serine Proteases

Serine proteases containing clip domains are implicated in PPO activation in arthropods [26]. To identify putative candidate proteases that might function as PAPs in *Drosophila*, we initially checked the sequences of 47 clip-domain serine proteases and serine protease homologs retrieved from the ImmunoDB. Among those, only 26 proteins possessed clip domain as well as a catalytic triad with the characteristics of active serine proteases. A phylogenetic analysis was then performed based on the deduced amino acid sequences of these 26 selected *Drosophila* sequences and others known to be involved in activation of melanization reaction in other species. As shown in Fig. S1 and Fig. S2, many of the clades in the phylogeny have low support. One clade, with a bootstrap value of 34, includes *Drosophila* MP2 (CG3066), *An. gambiae* CLIPB9 and *H. diomphalia* PPAF1. Another clade, with a bootstrap value of 52, groups *Drosophila* CG9737 with *M. sexta* PAP-2 and -3, and with *B. mori* PPAE. All of the proteases that cluster with *Drosophila* MP2 or CG9737 function as a PAP in initiating melanization [12], [18], [20], [25]. MP2 was reported to participate in melanization process in *Drosophila* [27], [28]. However, its substrate is still unknown and therefore we focused on it in the work reported here.

### MP2 Sequence Analysis

*Drosophila* MP2 consists of 391 amino acids residues including a predicted 27-residue secretion signal peptide. The calculated mass and isoelectric point of mature proMP2 are 41,260 Da and 6.11. There are nine potential *O*-linked glycosylation sites and one potential *N*-linked glycosylation site (Fig. S3). MP2 is composed of an amino-terminal clip domain connected by a linker region to a carboxyl-terminal catalytic domain containing three conserved amino acid residues: histidine, aspartic acid, and serine [45]. The predicted proteolytic activation site is located at FSNK^136^↓VYNG (Fig. S3). After cleavage, the resulting polypeptide fragments containing the clip domain and the catalytic domain would be expected to remain connected by an interchain disulfide bond (Cys^128^–Cys^264^).

### Production of Recombinant proMP2, proMP2_Xa_, Spn27A, and DmPPO1

**Figure 1:**
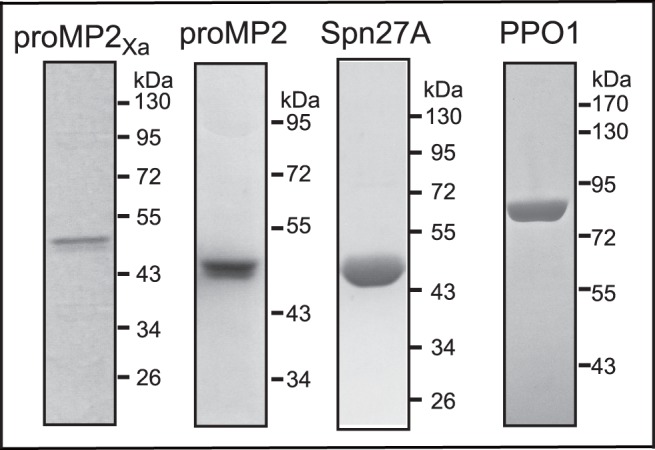
SDS-PAGE analysis of recombinant proMP2_Xa_, proMP2, Spn27A and PPO1. The purified recombinant proMP2_Xa_ (0.7 µg), proMP2 (0.99 µg) and Spn27A (2.6 µg) were treated with SDS sample buffer containing β-mercaptoethanol and separated by 10% SDS-PAGE followed by coomassie brilliant blue staining. Recombinant PPO1 (1.3 µg) was separated by 7.5% SDS-PAGE. The sizes and positions of the molecular mass standards are indicated on the *right*.

In order to investigate the roles of MP2 and Spn27A in *Drosophila*, we expressed and purified them as recombinant proteins. MP2 was expressed in zymogen form. To obtain the activated form of MP2, we mutated its predicted activation site to allow its activation by bovine Factor Xa. This mutant was named as proMP2_Xa_. We have used this approach previously to prepare zymogens that can be activated by the commercially available Factor Xa [10], [25]. Both recombinant proMP2 and proMP2_Xa_ were secreted from Sf9 cells using the MP2 secretion signal peptide. SDS-PAGE analysis indicated that proMP2 and proMP2_Xa_ had a similar apparent molecular mass of 49 kDa (Fig. 1), slightly larger than that predicted from the cDNA sequences (41.3 kDa). The increased mass is likely due to glycosylation, as the protein sequence contains ten potential glycosylation sites (Fig. S3). Spn27A and PPO1 were expressed in *E. coli*. SDS-PAGE analysis demonstrated that purified Spn27A and PPO1 had a mass of ∼48 and ∼79 kDa, respectively, similar to predicted mass based on their sequences (Fig. 1).

### Activation of Recombinant proMP2_Xa_ by Factor Xa

**Figure 2:**
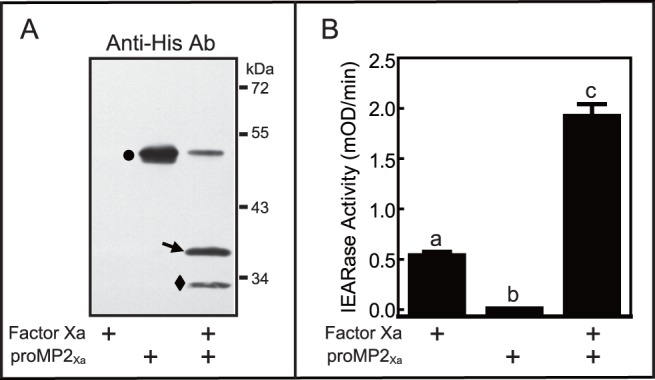
Activation of purified proMP2_Xa_ by bovine Factor Xa. (**A**) Purified recombinant proMP2_Xa_ (350 ng) was incubated with Factor Xa (200 ng) at 37°C for 1 h, and the mixtures were separated by 10% SDS-PAGE followed by immunoblot analysis using Anti-His antibodies (diluted 1∶1000). The sizes and positions of molecular weight standards are indicated on the *right*. Circle, arrow, and diamond indicated proMP2_Xa_, catalytic domain of proMP2_Xa_, and non-specific cleavage of proMP2_Xa_, respectively. (**B**) Amidase activity assay of activated MP2_Xa_ using IEAR*p*NA as a substrate, as described under “Materials and methods”. The bars represent mean ± S.D. (n = 3). *Bars* labeled with different letters (*a*, *b*, and *c*) are significantly different (analysis of one-way ANOVA followed by Newman-Keuls test, *P*<0.05).

To detect activation of proMP2_Xa_ by Factor Xa, we performed immunoblot analysis and amidase activity assays (Fig. 2). Incubation of purified proMP2_Xa_ with Factor Xa resulted in decreased intensity of the 49-kDa zymogen band and appearance of a 36-kDa band corresponding to the catalytic domain (Fig. 2A), as expected for activation. This result indicates that Factor Xa effectively cleaved proMP2_Xa_. In addition to the 36-kDa band, a band with apparent molecular weight of ∼30 kDa was detected by anti-His antibodies. This 30-kDa band might be due to the non-expected cleavage of proMP2_Xa_ by Factor Xa, as this band also appeared in the reaction mixture of Factor Xa and proMP2, which lacks IEGR sites.

To confirm the activation of proMP2_Xa_ by Factor Xa, we assayed for amidase activity of MP2_Xa_ using the colorimetric substrate IEAR*p*NA. A significant increase in activity (above that of Factor Xa alone) was observed in the presence of MP2_Xa_ that was activated by Factor Xa (Fig. 2B), thus confirming activation of proMP2_Xa_.

### MP2_Xa_ Directly Cleaves and Activates *Drosophila* PPO1 in Hemolymph and *in vitro*

**Figure 3:**
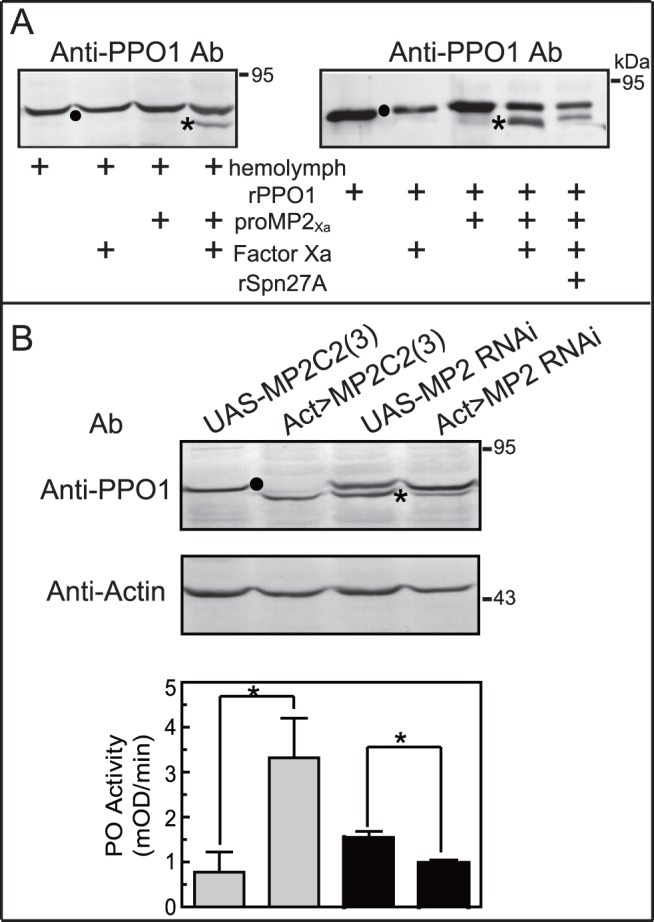
MP2 directly cleaves *Drosophila* PPO1. (**A**) MP2_Xa_ cleaves *Drosophila* PPO1 in hemolymph (*Left*) or as purified proteins (*Right*). Asterisks cleaved and activated PPO1; circle, PPO1 zymogen. (**B**) MP2 cleaves native PPO1 in transgenetic flies. Hemolymph was collected from flies with the overexpression of preactivated MP2 (*Act>MP2C2*) or depletion of MP2 (*Act-MP2RNAi*), and respective control flies. Samples were subjected to immunoblot analysis using Anti-PPO1 antibodies (*upper* panel) and PO activity assay (*lower* panel). The bars represent mean ± S.D. (n = 3). Asterisks indicate means that are significantly different from control (unpaired *t* test, *P*<0.05).

To characterize the role of MP2 in *Drosophila* PPO activation cascade, we incubated Factor Xa-activated MP2_Xa_ with hemolymph from *w* ^1118^ control flies. Immunoblot analysis, using *Drosophila* anti-PPO1 antibodies, identified a single band with the molecular weight of ∼90 kDa in hemolymph alone, which represented native *Drosophila* PPO1. No change was observed after incubation the hemolymph with Factor Xa or proMP2_Xa_ zymogen (Fig. 3A, left panel). However, when Factor Xa-activated MP2_Xa_ was incubated with the hemolymph, a band of ∼83 kDa, corresponding to active phenoloxidase-1, was detected (Fig. 3A, left panel), indicating that active MP2_Xa_ causes predicted cleavage of *Drosophila* PPO1.

We incubated active MP2_Xa_ with purified recombinant *Drosophila* PPO1 to explore the cleavage of this zymogen by MP2 *in vitro*. Antibodies against *Drosophila* PPO1 detected this recombinant protein as a main band with the apparent molecular weight of ∼90 kDa (Fig. 3A, right panel). No change was observed in the mixtures containing recombinant PPO1 and proMP2_Xa_ zymogen but incubation of the recombinant PPO1 with Factor Xa-activated MP2_Xa_ resulted in decreased intensity of the 90 kDa zymogen and the appearance of an immunoreactive band at ∼83 kDa, the expected size for activated phenoloxidase-1 (Fig. 3A, right panel). Pre-incubation of Spn27A and MP2_Xa_ diminished the intensity of this band, suggesting that Spn27A prevented MP2 from cleaving *Drosophila* PPO1.

To further investigate the roles of MP2 in melanization in flies, hemolymph from flies with either overexpression (*Act*>*MP2C2*) or repression (*Act*>*MP2 RNAi*) of MP2 was collected for immunoblot analysis and phenoloxidase activity assay. Anti-PPO1 antibodies recognized a ∼90-kDa band corresponding to PPO in control hemolymph (*UAS-MP2C2*). This band disappeared, and an immunoreactive band at ∼83-kDa (the expected active band of phenoloxidase-1) was observed in hemolymph overexpressing pre-activated MP2 (*Act>MP2C2*) (Fig. 3B, upper panel). The phenoloxidase activity of this hemolymph increased significantly (Fig. 3B, lower panel). Conversely, the intensity of the ∼83-kDa band decreased, compared to controls (*UAS-MP2 RNAi*), in hemolymph from flies lower expressing MP2 (Fig. 3B, upper panel), and phenoloxidase activity decreased significantly (Fig. 3B, lower panel).

### Spn27A Inhibits MP2 Activity

**Figure 4:**
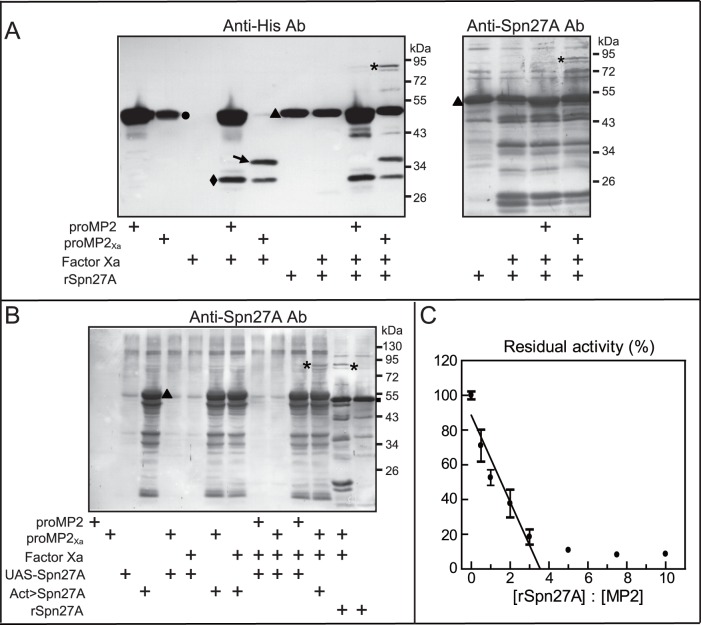
*Drosophila* Spn27A binds and inhibits MP2. SDS-stable complex formation between MP2_Xa_ and recombinant Spn27A (**A**) or native Spn27A in *Drosophila* hemolymph (**B**). ProMP2_Xa_ (350 ng) was activated by Factor Xa and then incubated for 30 min at room temperature with 1-fold molar of recombinant Spn27A or 3 µl of hemolymph. In control reactions, proMP2_Xa_ or Factor Xa was omitted, or proMP2 was used instead. The samples were subjected to SDS-PAGE and immunoblot analysis using mouse anti-His (*Left*) or rabbit anti-Spn27A (*Right*) antibodies. The notes used in the figure were: circle, proMP2_Xa_; arrow, catalytic domain of proMP2_Xa_; diamond, non-specific cleavage of proMP2_Xa_; triangles, non-complexed Spn27A; asterisks, Spn27A-MP2_Xa_ complex. (**C**) Stoichiometry of inhibition of MP2 by Spn27A. Recombinant Spn27A was incubated with Factor Xa-activated MP2_Xa_ at different molar ratios for 30 min at room temperature. The residual amidase activity was measured using IEAR*p*NA as substrate, and plotted as mean ± S.D. (n = 3) against the corresponding molar ratios of Spn27A and MP2_Xa_.

The formation of an SDS-stable complex of serpin with a protease it inhibits is a characteristic feature of a serpin-protease reaction [33]. As a first step to determine if MP2 can be directly inhibited by Spn27A, we tested whether these two proteins form such a complex *in vitro*. Purified recombinant Spn27A and Factor Xa-activated MP2_Xa_ were combined for SDS-PAGE and immunoblotting with anti-His or anti-Spn27A antibodies (Fig. 4A). Anti-His antibodies recognized the 49-kDa MP2_Xa_ zymogen and 48-kDa recombinant Spn27A, which could not be separated on 10% SDS-PAGE, and the 36-kDa active MP2_Xa_ (Fig. 4A, left panel). When Spn27A was combined with active MP2_Xa_, the intensity of the 36-kDa band corresponding to the MP2 catalytic domain decreased and an immunoreactive band at ∼80-kDa (the expected size of the Spn27A/MP2_Xa_ complex) appeared. This band was also recognized by anti-Spn27A antibodies (Fig. 4A, right panel). We also expressed another recombinant serpin, Spn77Ba, which was also reported to regulate melanization in *Drosophila* [39]. When Factor Xa-actived MP2_Xa_ was mixed with Spn77Ba, no band with apparent high molecular weight (∼80-kDa) was detected by Anti-His antibodies (Fig. S4). These results suggest that the formation of covalent complexes between MP2 and Spn27A is specific and that Spn27A is an inhibitor of MP2.

To test if this complex can be formed *in vivo*, we combined *Drosophila* hemolymph and recombinant MP2_Xa_. Since endogenous Spn27A in wild-type flies might be not abundant, we collected hemolymph from flies overexpressing Spn27A (*act>Spn27A*). Hemolymph collected from adult flies bearing UAS-Spn27A constructs were used as controls. Native Spn27A in the hemolymph was detected at the position of 48 kDa by anti-Spn27A antibodies. This 48-kDa band was faint in UAS-Spn27A control flies, but it became strong after Spn27A was over-expressed via actin driver. When Factor Xa-activated MP2_Xa_ was mixed with such hemolymph, a higher molecular weight band of ∼80 kDa in addition to the 48-kDa Spn27A band was detected by anti-Spn27A antibodies (Fig. 4B). This 80-kDa band was at the same position as complexes formed between recombinant MP2_Xa_ and recombinant Spn27A. Anti-Spn27A antibodies failed to detect any band corresponding to the SDS-stable Spn27A-MP2 complex when active MP2_Xa_ was mixed with control hemolymph (*UAS-Spn27A*) without the overexpression of Spn27A (Fig. 4B).

To further investigate the inhibition of MP2_Xa_ by Spn27A, we tested Spn27A’s ability to inhibit hydrolysis of a colorimetric peptide substrate by MP2_Xa_. MP2_Xa_ activity decreased linearly as Spn27A concentration increased (Fig. 4C). The stoichiometry of inhibition was 3.5, indicating that under the experimental conditions Spn27A preferentially acts as an inhibitor rather than a substrate of MP2 [33].

## Discussion

Proteolytic activation of PPO is a critical step in the host defense system against invading pathogens and parasites and, as such, has been extensively investigated in various insects and crustaceans for more than 40 years [46]. Even so, understanding of the PPO activation cascade is still incomplete. Especially in *Drosophila*, current knowledge of this cascade is still limited. Studies of the PPO activation cascade in several relatively large insects, in which biochemical studies were feasible, have established the basic outlines of this cascade as well as aspects of its control by serpins. Those pioneering studies have set the scene for continued studies in other insects in which the initial biochemical work would have been very difficult. We suggest that *Drosophila* is especially appropriate for such continued studies, because of the wide range of genetic and molecular genetic tools available in that system. In combination with the expression of recombinant proteins for biochemical studies, these genetic and molecular genetic tools will allow deeper understanding of PPO activation cascade in *Drosophila*. Furthermore, although PPO activation cascade does not exist in mammals, it is analogous to other protease-cascades in humans, especially complement activation and blood coagulation. As regards the roles of serpins in regulations of protease-cascades, we can expect that insights from studies in *Drosophila* and other insects will be relevant to better understand the control of analogous cascades in mammals. Therefore, in this research we combined biochemical and genetic techniques and investigated the molecular mechanisms of PPO activation in *Drosophila*.

Sequence analysis demonstrated that there are 26 genes encoding clip-domain serine proteases in the *Drosophila* genome (Fig. S1, Fig. S2 and Ref [47]). Among them, in our phylogenetic tree MP2 is clustered with *An. gambiae* CLIPB9 and *H. diomphalia* PPAF1, which are both known to function in melanization (Fig. S1 and Ref [24], [25]). MP2 has been suggested to be involved in PPO activation because overexpression of activated MP2 resulted in constitutive melanization in larvae, pupae, and adults in *Drosophila*, whereas lack of MP2 produced a failure to activate melanization upon microbial challenge [27], [28]. Therefore, MP2 was a good candidate to function in PPO activation. We therefore performed, and report here, *in vitro* and *in vivo* experiments to determine whether MP2 directly activates PPO. The addition of active MP2 to *Drosophila* hemolymph did result in cleavage of PPO1, and active MP2 also directly cleaved and activated recombinant *Drosophila* PPO1 *in vitro* (Fig. 3A). When the expression of MP2 was elevated or knocked down using Gal4/UAS system, the cleavage of PPO1 and the level of PO activity in hemolymph increased or decreased accordingly (Fig. 3B). These results suggest that MP2 functions as the terminal protease in the PPO activation pathway.

**Figure 5:**
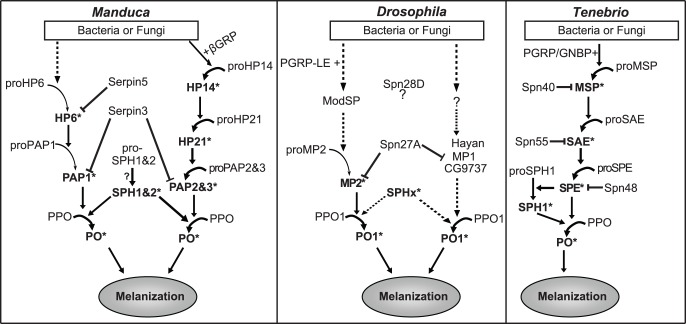
A model for activation of melanization in *Drosophila* and comparison with *M. sexta* and *T. molitor* melanization pathway [10]–[14], [18], [28], [29], [34], [37], [47], [49], [60], [61]. *Arrows* indicate activation of downstream components or steps. *Dashed arrows* indicate steps that have not been experimentally verified. Regulation of proteases by serpins is indicted.

It is notable that residual non-cleavage of PPO1 remained detectable and low phenoloxidase activity still existed in flies expressing MP2 dsRNA for transcript knockdown via RNAi (Fig. 3B). A possible reason could be incomplete knockdown of MP2, potentially allowing for residual MP2 activity in *Drosophila*. Quantitative RT-PCR analysis showed that the transcript levels in MP2 repressing flies were reduced to roughly 45% (Fig. S5). Another probable explanation is the existence of other PAPs in *Drosophila* which share this function with MP2. It is conceivable that several PAPs work cooperatively to activate melanization reaction in one insect. For example, three such enzymes (named PAP1, PAP2, and PAP3) can cleave and activate PPO in *M. sexta* [12], [18], [20], although they themselves are activated by different serine proteases [10], [11], [19]. In *Drosophila*, Hayan has been identified as a PAP [29]. In addition, we identified in the *Drosophila* genome MP2 is most similar to *M. sexta* PAP1, and CG9737 has the highest similarity to *M. sexta* PAP2 and PAP3 (Fig. S1) in 26 clip-containing serine proteases. We therefore hypothesize that *Drosophila* CG9737 may also act as a PAP, and that its zymogen is activated by a protease different from that one activating proMP2. Tang *et al* reported MP1 and MP2 defined a melanization cascade in *Drosophila*, and MP1 is required to activate the melanization in response to both bacterial and fungal infection while MP2 is mainly involved in the infection of fungi [28]. MP1 potentially acts as a PAP to activate the PPO zymogen [28]. Based on the results presented in this study, we hypothesize that there exist multiple PAPs in *Drosophila*, and MP1, MP2, Hayan, and CG9737 could well be involved in different branches and respond to the infection by different types of pathogens (Fig. 5). We tried several expression systems to obtain recombinant MP1 with activity, but failed. Once the recombinant active MP1 is available, we will investigate the potential role of MP1 in activating the melanization cascade, and the possible relationship between MP1 and MP2.

Extracellular serine protease cascades, including the PPO activation cascade in arthropods and the blood coagulation cascade in horseshoe crabs and humans, are regulated in order to prevent unnecessary or undesirable activation and to limit reactions to an appropriate length of time and to appropriate discrete locations [31], [32]. This regulation is performed, at least in part, by inhibitors of the serpin superfamily [31], [32]. In *Drosophila*, genetic analysis has demonstrated that Spn27A and Spn28D regulate PPO activation pathway in hemolymph [36]–[38], and Spn77Ba and Spn28D control melanization in the trachae [38], [40]. The work we report here reveals that *Drosophila* Spn27A inhibits MP2 from cleaving PPO and forms SDS-stable complexes with recombinant and endogenous MP2 (Fig. 3 and 4). Overexpression of Spn27A blocked cleavage of PPO1 and reduced phenoloxidase activity significantly (Fig. S6). A previous report also indicated Spn27A forms a complex with exogenous PAP from *H. diomphalia*, and restricts PO activity to the site of injury or infection [36], [37].

We note that cleavage of PPO-1 was completely blocked in flies overexpressing Spn27A (Fig. S6), but was only partially inhibited in MP2 repressing flies (Fig. 3B). This may be due to the incomplete depletion of MP2, since the knockdown efficiency of MP2 in transgenetic flies was around 45% (Fig. S5). An equally parsimonious explanation is that Spn27A has other protease targets besides MP2. Arthropod and also mammalian serpins commonly have several physiological protease targets. For example, *M, sexta* serpin-3 inhibits both PAP1 and PAP3 [34], serpin-1J inhibits both PAP3 and HP8 [18], [48], and human anti-thrombin III inhibits not only thrombin, but also Factor Xa and several other plasma proteases [49]. Leclerc *et al* observed that *Spn27A*; *MP2^KG02818^* double-mutant flies presented spontaneous large melanization spots, with the same frequency as *Spn27A* single mutants [27]. They speculated that MP2 did not directly cleave PPO, but instead activated a downstream PAP which was also regulated by Spn27A. Based on the results from our experiments and the literature, we propose the following hypothesis to interpret the above observations: Spn27A inhibits MP2 and another PAP (or proteases). When MP2 is inactivated by insertion of the P element KG02818, another (redundant) PAP (or proteases) such as Hayan or MP1 compensates for the deficiency of MP2. Hayan is involved in Spn27A-controlled PO activity, but not inhibited directly by Spn27A [29]. However, it is highly possible that MP1 is the direct target protease of Spn27A, because induction of PO activity by overexpression of preactivated MP1 was blocked by simultaneous overexpression of Spn27A [28]. Experiments are underway to study the possible regulation of Spn27A on MP1.

In this study, we identified *Drosophila* MP2 as a PAP, and found that MP2 is inhibited by Spn27A in activating melanization. Based on our work reported here and other relevant observations as cited above, we propose a model for activation and regulation of melanization in *Drosophila*. Upon bacterial or fungal infection, specific patterns on the surface of invading pathogens are recognized by pattern recognition protein, such as PGRP-LE [50]. Then, an unknown, modular serine protease is activated in the presence of pattern recognition protein. Once activated, this unknown protease processes a series of clip-domain serine proteases, which leads to activation of proMP2. Active MP2 then directly cleaves PPO to produce active PO (Fig. 5). In another activation branch, Hayan or MP1 or CG9737 functions as a distinct PAP. Spn27A inhibits MP2 and other PAPs. Spn28D does not inhibit MP2, but confines PO availability by controlling its initial release [38].

In addition, we also note that the efficiency of cleavage of PPO1 by MP2 *in vitro* was relatively low, although it did occur (Fig. 3A). Perhaps a protein in addition to MP2 is required *in vivo* for PPO activation. In fact the required involvement of proteins other than PAPs for PPO activation has been demonstrated in *Hyalophora cecropia*, *H. diomphalia*, *M. sexta*, and *T. molitor* [13], [51]–[53]. Clip-domain serine protease homologs whose catalytic serines are replaced with glycine and therefore lack proteolytic activity are required for generating active PPO, enhancing the cleavage by PAPs. We therefore suggest that also in *Drosophila* serine-protease homologs function as “protein cofactors” to facilitate PPO activation. Bioinformatic analysis indicates that there are 19 genes for serine-protease homologs in *Drosophila* [47]. Experiments to examine their functions are underway in our laboratory.

## Materials and Methods

### *Drosophila* Stocks

Flies were maintained on standard cornmeal-yeast medium at 25°C under a relative humidity of 60% and a photoperiod of 12 h light. Fly strain *w* ^1118^ was used as the wild-type control unless otherwise specified. Other transgenic fly strains used were as follows: *act-Gal4*, *UAS-Spn27A* [36], *UAS-MP2C2*(3) [28], *UAS-MP2Ri1*(3) [28]. Transgenes were expressed using the Gal4-UAS system [54].

### Phylogenetic Analysis of Serine Proteases in *Drosophila*

To identify serine proteases that are potentially involved in melanization in *Drosophila*, protein sequences of all annotated, putatively active serine proteases were retrieved from the *Drosophila* genome (http://cegg.unige.ch/Insecta/immunodb). Their catalytic-domain sequences were aligned with serine proteases from other species known to have a function related to melanization using the Clustal W program. Phylogenetic trees were constructed by the neighbor-joining method using MEGA version 4 software [55]. For the neighbor-joining method, gaps were treated as characters, and statistical analysis was performed by the bootstrap test, with 1,000 repetitions. The sequences (with GenBank accession number) used for the alignment with *Drosophila* serine proteases were: *B. mori* BAEEase (ABB58762) and PPAE (NP_001036832); *H. diomphalia* PPAF1 (BAA34642); *M. sexta* HP6 (AAV91004), HP8 (AAV91006), HP13 (AAV91011), HP15 (AAV91012), HP21 (AAV91019), PAP1 (AAX18636), PAP2 (AAL76085), and PAP3 (AAO74570); *T. molitor* SAE (AB363979) and SPE (AB363980).

### Preparation of Recombinant proMP2, proMP2_Xa_, Spn27A and PPO-1

To biochemically characterize roles and regulation of MP2 in *Drosophila* melanization, we expressed recombinant proMP2, Spn27A, and PPO-1 using baculovious or prokaryote system. For the production of proMP2, primers (Table S1) designed based on the nucleotide sequences in FlyBase were used to amplify the full length of proMP2 from cDNA of adult flies. The PCR product was recovered and ligated to pMD19-T vector. The resulting plasmid was used as template to amplify the entire proMP2 coding region, including the signal peptide, using primers listed in Table S1. The forward primer included a *Spe*I site, and the reverse primer contained three codons for glycine and six codons for histidine residues followed by a stop codon and a *Hind*III site. The PCR product was recovered by agarose gel electrophoresis, digested with *Spe*I and *Hind*III and then inserted into the corresponding restriction sites in the vector pFastBac1 (Invitrogen). The resulting proMP2 plasmid, after sequence confirmation, was used as template to produce mutant proMP2 (proMP2_Xa_) plasmids following Chiu’s methods [56]. In proMP2_Xa_, residues 133–136 at the predicted activation site of proMP2 were changed from FSNK^136^ to IEGR^136^ to permit its activation by bovine Factor Xa [57]. After sequence verification, proMP2 and proMP2_Xa_ plasmids were used to generate a recombinant baculovirus using Cellfectin® II Reagent (Invitrogen). To express proMP2 and proMP2_Xa_, Sf9 cells (2×10^6^ cells/ml) in 500 ml of Insect-Xpress protein-free medium (Lonza) were infected with the recombinant baculovirus at multiplicity of infection of 5 and were incubated at 28°C with shaking at 150 rpm. The culture was harvested at 48 h post infection, and cells were removed by centrifugation at 5000×*g* for 20 min at 4°C. The cell-free medium was used to further purify recombinant proMP2 and proMP2_Xa_ following the method described previously [58].

To express Spn27A, a cDNA fragment encoding mature Spn27A was amplified by PCR using gene-specific primers (seen in Table S1). The forward primer contained an *Nco*I restriction site, which provided the start codon, followed by six codons for histidine residues. The reverse primer included an *Xho*I site at the 3' end of stop codon. The PCR product was cloned into pMD19 T-vector. The resulting plasmid was used as template to produce silent mutated plasmids with Chiu’s methods [56]. In plasmids with silent mutation, nucleotides 965–970 were changed from CCATGG to CTATGG, which prevented digestion by *Nco*I but retained the original amino acid sequence. After sequence verification, the mutant plasmids were excised with *Nco*I and *Xho*I and subcloned into the same restriction sites in the expression vector pET-28a (Novagen). The resulting plasmid was used to transform *E. coli* BL21 (DE3) strain. For recombinant Spn27A expression, these bacteria were grown at 37°C in LB medium containing 50 µg/ml of kanamycin. When OD_600_ of the culture reached 0.8, isopropyl β-D-thiogalactoside was added to a final concentration of 0.1 mM, and the recombinant protein was expressed for 10 h at 25°C. The bacteria were harvested by centrifugation at 4500 *g* for 20 min, and resuspended in lysis buffer (50 mM sodium phosphate, 300 mM NaCl, 10 mM imidazole, pH 8.0). After being lysed by sonication, soluble Spn27A in the cleared lysate was purified by nickel-nitrilotriacetic acid (NTA) agarose chromatography as described by An *et al* [58].

For expressing recombinant *Drosophila* PPO-1, plasmids in which a fragment encoding full length of prophoneoloxidase-1 was inserted into the restrictions sites *Nco*I and *Nde*I in vector pET28b (Novagen) were kindly provided by Dr. Erjun Ling (Institute of Plant Physiology and Ecology, Chinese Academy of Sciences), and used to transform *E. coli* strain BL21(DE3). The protein was then expressed and purified following the methods described by Li *et al* [59].

### SDS-polyacrylamide Gel Electrophoresis (SDS-PAGE) and Immunoblot Analysis

Protein concentrations were determined using Bradford Reagent Solution (Sangon) with bovine serum albumin as a standard. For SDS-PAGE, protein samples were treated with 5× SDS sample buffer containing β-mercaptoethanol at 95°C for 5 min and then separated by 10% SDS-PAGE. Proteins were detected by staining with coomassie brilliant blue. For immunoblot analysis, proteins were transferred onto a nitrocellulose membrane and detected with mouse anti-His (1∶1000), or rabbit anti-*Drosophila* PPO1 (1∶2,000, generously provided by Dr. Erjun Ling) as primary antibodies. Antibody binding was visualized using alkaline phosphate-conjugated goat anti-mouse or anti-rabbit IgG (diluted 1∶2000) and 5-Bromo-4-chloro-3-indolyl phosphate/Nitro blue tetrazolium (BCIP/NBT) staining buffer containing 165 µg/ml BCIP and 330 µg/ml NBT in 100 mM Tris (pH 9.5), 150 mM NaCl, and 5 mM MgCl_2_.

### Activation of Recombinant proMP2_Xa_ by Factor Xa

To test whether proMP2_Xa_ could be activated by Factor Xa, 350 ng of purified recombinant proMP2_Xa_ was incubated with 200 ng of bovine Factor Xa (New England Biolabs) in the reaction buffer (20 mM Tris-HCl, pH 8.0, 150 mM NaCl, 2 mM CaCl_2_, pH8.0) at 37°C for 1 h. The mixtures were separated by 10% SDS-PAGE followed by immunoblot analysis. The activation of proMP2_Xa_ was confirmed by measuring the amidase activity of activated MP2_Xa_ with 200 µl of 50 µM acetyl-Ile-Glu-Ala-Arg-*p*-nitroanilide (IEAR*p*NA) in 0.1 M Tris-HCl (pH8.0), 0.1 M NaCl and 5 mM CaCl_2_ as colorimetric substrate. The amidase activity was measured by monitoring *A*_405_ in a microplate reader (Bio-Tek Instrument, Inc.). One unit of amidase activity was defined as Δ*A*_405_/min = 0.001.

### PPO Activation Assays

To investigate the role of MP2 in activating *Drosophila* PPO-1 *in vitro*, 350 ng of proMP2_Xa_ or Factor-Xa activated MP2_Xa_ were incubated with 380 ng of purified recombinant PPO-1 or 3.5 µl of hemolymph from *w* ^1118^ adult flies at 37°C. After one hour, reaction mixtures were subjected to 7.5% SDS-PAGE and immunoblot analysis.

To measure the ability of MP2 to activate PPO-1 *in vivo*, hemolymph was collected from adult flies bearing the catalytic domain of MP2 or of MP2 RNAi constructs. Respective control flies were also used for hemolymph collection simultaneously. In order to collect the hemolymph from flies with MP2 RNAi constructs, adult flies were pierced with a needle of 0.1 mm diameter previously dipped into a concentrated *E. coli* culture [28]. Samples were subjected to SDS-PAGE and immunoblot analysis. Additionally, phenoloxidase activity in the collected hemolymph was measured using dopamine as substrate [10]. One unit of phenoloxidase activity was defined as the amount of enzyme producing an increase in absorbance (ΔA_470_) of 0.001 per min.

### Detection of SDS-stable Complexes Between MP2_Xa_ and Spn27A

To detect the formation of covalent complexes between MP2 and Spn27A *in vitro*, recombinant proMP2_Xa_ (350 ng) was activated by Factor Xa as described above and mixed with purified Spn27A at a molar ratio of 1∶1. In control samples, proMP2_Xa_ or Factor Xa was omitted from the mixture. After incubation at room temperature for 30 min, the reaction mixtures were subjected to 10% SDS-PAGE and immunoblot analysis.

To detect formation of the MP2-Spn27A complex *in vivo*, hemolymph (insect blood) from 15 one-day old *UAS-Spn27A* or *act>Spn27A* adult flies was collected into phosphate-buffered saline as described [28], and mixed with 200 ng of Factor Xa-activated MP2_Xa_. After incubation at 37°C for 30 min, samples were subjected to SDS-PAGE and immunoblot analysis.

### Inhibition of MP2_Xa_ Activity by Spn27A

To assess the inhibitory potential of Spn27A towards MP2, proMP2_Xa_ (360 ng) was activated by Factor Xa and mixed with recombinant Spn27A at different molar ratios (0.5∶1 to 10∶1) in 30 µl of buffer (150 mM NaCl, 20 mM Tris, pH8.0). In control reactions, the same amount of Factor Xa used to activate proMP2_Xa_ was substituted for active MP2_Xa_. After incubation at room temperature for 30 min, residual amidase activity was measured as described above. Amidase activity of MP2_Xa_ was defined as the activity of MP2_Xa_ minus the activity of Factor Xa alone.

## Supporting Information

Figure S1 **Phylogenetic analysis of the catalytic domains in clip-domain serine proteases from** ***Drosophila*** **and other insect species.** The tree was derived from sequence alignment using Clustal W. Numbers at the nodes indicate the bootstrap confidence values of 1000 replicates. The scale bar indicates the number of substitutions per site. A very similar tree was obtained when the clip domains were included in the alignment. The circled bootstrap values indicate two clades that group *Drosophila* MP2 or CG9737 with other proteases involved in melanization reactions, respectively. Sequence identifiers for *Drosophila* proteases include Flybase number followed by a name given in the references if this protease has been studied functionally. The other sequences used for the analysis were: *Anopheles gambiae* CLIPB8, CLIPB9 and CLIPB10; *Bombyx mori* BAEEase and PPAE; *Holotrichia diomphalia* PPAF1; *Manduca sexta* HP6, HP8, HP13, HP15, HP21, PAP1, PAP2, and PAP3; *Tenebrio molitor* SAE and SPE.
(EPS)

Figure S2 **Sequences used in alignment of the catalytic domains in clip-domain serine proteases from** ***Drosophila*** **and other insect species.** Totally forty-two amino acid sequences were aligned for estimating the tree shown in FigureS1. Sequences were retrieved from ImmunoDB (http://cegg.unige.ch/Insecta/immunodb) and checked manually. Species acronym is defined as follows: *Anopheles gambiae*, Ag; *Bombyx mori*, Bm; *Holotrichia diomphalia*, Hd; *Manduca sexta*, Ms; *Tenebrio molitor*, Tm. *Drosophila* proteases are indicated by the corresponding Flybase numbers.
(PDF)

Figure S3 **Sequence analysis of** ***Drosophila*** **MP2.** The deduced amino acid sequences of MP2 are retrieved from GenBank™. The predicted secretion signal peptide is double-underlined. The proteolytic activation site is indicated with “↓”. Three amino acid residues (Histidine, Aspartic acid, and Serine) critical for the catalytic activity of MP2 are shown in bold. Putative N-linked and O-linked glycosylation sites are shaded. Clip domain, linker region, and catalytic domain are shown. The absolutely conserved cysteines in clip domain are bold-italicized and numbered. The paired numbers (1–1, 2–2, 3–3) indicate the intramolecular disulfide linkage. Another two cysteines with numbers (4–4) indicate the disulfide linkage between clip domain and catalytic domain, which remains two domains connected after cleavage at the activation site. Nine amino acid residues (*GGGHHHHHH*) added to the carboxyl terminal for the expression of recombinant MP2 are italicized.
(EPS)

Figure S4 **No complex formed between MP2_Xa_ and Spn77Ba.** ProMP2_Xa_ (350 ng) was activated by Factor Xa and then incubated with Spn77Ba (450 ng) for 30 min at room temperature. In control reactions, proMP2_Xa_ or Factor Xa was omitted. The samples were subjected to 10% SDS-PAGE and immunoblot analysis using anti-His antibodies (diluted 1∶1000). Size and positions of molecular mass standard are indicated to the *right* of the blot. No band with high molecular weight, consistent with the expected size for a serpin-protease complex, was recognized by anti-His antibodies. It suggests that MP2_Xa_ has no potential to form SDS-stable complex with Spn77Ba.
(EPS)

Figure S5 **MP2 knockdown efficiency in MP2 repressing flies.** Expression levels were measured by quantitative RT–PCR, using ribosomal protein S49 mRNA levels for normalization and UAS-MP2 RNAi samples as the calibrator.
(EPS)

Figure S6 **Detection of cleavage of PPO1 (A) and PO activity (B) in flies over-expressing Spn27A.** Transgenetic flies were generated by combing one copy of a UAS-*Spn27A* construct with one copy of act-*Gal4* driver. Hemolymph was collected from Act-*Spn27A* transgenetic flies and UAS-*Spn27A* control flies, and then subjected to immunoblot analysis using Anti-PPO1 antibodies **(A)** and PO activity **(B)**. Circle, PPO1 zymogen; arrow, cleaved and activated PPO. The bars represent mean ± S.D. (n = 3). Asterisks indicate means that are significantly different from control (unpaired *t* test, *P*<0.05).
(EPS)

Table S1 **Primer sequences.**
(EPS)
