# Supplementary material for: Serine Protease MP2 Activates Prophenoloxidase in the Melanization Immune Response of Drosophila melanogaster
Source: PLoS One. 2013 Nov 15;8(11):e79533. doi: 10.1371/journal.pone.0079533 (PMC3829845; doi:10.1371/journal.pone.0079533)
Supplement: Figure S2 — Sequences used in alignment of the catalytic domains in clip-domain serine proteases from Drosophila and other insect species. Totally forty-two amino acid sequences were aligned for estimating the tree shown in FigureS1. Sequences were retrieved from ImmunoDB (http://cegg.unige.ch/Insecta/immunodb) and checked manually. Species acronym is defined as follows: Anopheles gambiae, Ag; Bombyx mori, Bm; Holotrichia diomphalia, Hd; Manduca sexta, Ms; Tenebrio molitor, Tm. Drosophila proteases are indicated by the corresponding Flybase numbers. (PDF) [file pone.0079533.s002.pdf]

Fig.S2

>CG5896
LSQRVSNGYEVKLSSRPWMALLRYQQFGESRFLCGGAMISERYILTAAHCVHGLQNDLYEIRLGE
HRISTEEDCRQQGRKKKCAPPVVNVGIEKHLIHEKYDARHIMHDIALLKLNRSVPFQKHIKPICL
PITDELKEKAEQISTYFVTGWGTTENGSSSDVLLQANVPLQPRSACSQAYRRAVPLSQLCVGGGD
LQDSCKGDSGGPLQAPAQYLGEYAPKMVEFGIVSQGVVTCGQISLPGLYTNVGEYVQWITDTMAS
NGL

>CG4920 (Easter)
LSNRIYGGMKTKIDEFPWMALIEYTKSQGKKGHHCGGSLISTRYVITASHCVNGKALPTDWRLSG
VRLGEWDTNTNPDCEVDVRGMKDCAPPHLDVPVERTIPHPDYIPASKNQVNDIALLRLAQQVEYT
DFVRPICLPLDVNLRSATFDGITMDVAGWGKTEQLSASNLKLKAAVEGFRMDECQNVYSSQDILL
EDTQMCAGGKEGVDSCRGDSGGPLIGLDTNKVNTYYFLAGVVSFGPTPCGLAGWPGVYTLVGKYV
DWIQNTIES

>CG1102 (MP1)
FGDRVVGGNETTKREFPWMALIEYTKPGNVKGHHCGGSLINHRYVLTAAHCVSAIPSDWELTGVR
LGEWDASTNPDCTVGKNGRRDCNEPYVDYPVEERIPHPQYPGNSRDQLNDIALLRLRDEVQYSDF
ILPVCLPTLASQHNNIFLGRKVVVAGWGRTETNFTSNIKLKAELDTVPTSECNQRYATQRRTVTT
KQMCAGGVEGVDSCRGDSGGPLLLEDYSNGNSNYYIAGVVSYGPTPCGLKGWPGVYTRVEAYLNW
IENNVRA

>CG7996 (Snake)
SVPLIVGGTPTRHGLFPHMAALGWTQGSGSKDQDIKWGCGGALVSELYVLTAAHCATSGSKPPDM
VRLGARQLNETSATQQDIKILIIVLHPKYRSSAYYHDIALLKLTRRVKFSEQVRPACLWQLPELQ
IPTVVAAGWGRTEFLGAKSNALRQVDLDVVPQMTCKQIYRKERRLPRGIIEGQFCAGYLPGGRDT
CQGDSGGPIHALLPEYNCVAFVVGITSFGKFCAAPNAPGVYTRLYSYLDWIEKIAFKQH

>CG6367 (Psh)
LVIHIVGGYPVDPGVYPHMAAIGYITFGTDFRCGGSLIASRFVLTAAHCVNTDANTPAFVRLGAV
NIENPDHSYQDIVIRSVKIHPQYVGNKYNDIAILELERDVVETDNIRPACLHTDATDPPSNSKFF
VAGWGVLNVTTRARSKILLRAGLELVPLDQCNISYAEQPGSIRLLKQGVIDSLLCAIDQKLIADA
CKGDSGGPLIHELNVEDGMYTIMGVISSGFGCATVTPGLYTRVSSYLDFIEGIVWPDNRV

>CG6361
LTVHILDGERVDRGVYPHMAAIAYNSFGSAAFRCGGSLIASRFVLTAAHCVNSDDSTPSFVRLGA
LNIENPEPGYQDINVIDVQIHPDYSGSSKYYDIAILQLAEDAKESDVIRPACLYTDRSDPPANYK
YFVAGWGVMNVTNRAVSKILLRAALDLVPADECNASFAEQPSANRTLRRGVIASQLCAADKNQRK
DACQGDSGGPLILEIDDVDGTYSIVGVISSGFGCATKTPGLYTRVSSFLDYIEGIVWPSNRF

>CG2056 (Spirit)
FFVSVVGGMPTRPREFPFMAALGWRSNFDQRIYYRCGGALIANNFVLTAAHCADLGGEPPSQVRL
GGDNLTLTEGEDISIRRVIIHPDYSASTAYNDIALLELETAAKPELKPTCIWTQKEVTNTLVTAI

GYGQTSFAGLSSAQLLKVPLKSVSNEECQHHYQKDQLAQGVLGTQMCAGDITGERDTCQGDSGGP
LLMQDGLLGYVVGITSLGQGCASGPPSVYTRVSSFVDWIEGIVWPAQQVTNAPQPNQMTSFSPEF
DLRATI

>CG9372
QFPRLTGGRPAEPDEWPWMAALLQEGLPFVWCGGVLITDRHVLTAAHCIYKKNKEDIFVRLGEYN
THMLNETRARDFRIANMVLHIDYNPQNYDNDIAIVRIDRATIFNTYIWPVCMPPVNEDWSDRNAI
VTGWGTQKFGGPHSNILMEVNLPVWKQSDCRSSFVQHVPDTAMCAGFPEGGQDSCQGDSGGPLLV
QLPNQRWVTIGIVSWGVGCGQRGRPGIYTRVDRYLDWILANADV

>CG31728
DQERIVGGINASPHEFPWIAVLFKSGKQFCGGSLITNSHILTAAHCVARMTSWDVAALTAHLGDY
NIGTDFEVQHVSRRIKRLVRHKGFEFSTLHNDVAILTLSEPVPFTREIQPICLPTSPSQQSRSYS
GQVATVAGWGSLRENGPQPSILQKVDIPIWTNAECARKYGRAAPGGIIESMICAGQAAKDSCSGD
SGGPMVINDGGRYTQVGIVSWGIGCGKGQYPGVYTRVTSLLPWIYKNIK

>CG4927
TTPFIVGGAKAAGREFPFMALLGQRGKNSSQIDWDCGAIIIHPKFVLTAAHCLETSETKEQRLDP
NYDGPKYVVRLGELDYNSTTDDAQPQDFRVLNYVVHPAYGEDDDTGSRKNDIAVVELEMEATFSE
YVAPACLPLDGGNEQLQVAAAGWGATSESGHASSHLLKVSLDRYDVAECSQRLEHKIDVRTQLCA
GSRSTSADTCYGDSGGPVFVQHPIYSCLKQVIGITSYGLVCGVQGLPSVYTKVHLYTDWIENIVW
GE

>CG3700
STPFIVGGTKASGKEFPFMALIGTHRPNKSKSDINWDCGGSVVHPKFVLTAAHCLETDESKAERL
DPNFDSPKFVVRLGELDYNSTTDDALVQDFRVVNYVVHPGYDTEDEEQGFKNDIALVELDRKAEF
NDHVAAVCLPPDSGNDVQQVTAAGWGFTADGVKSSHLLKVNLQRFSDEVCQKRLRFSIDTRTQFC
AGSMSSQADTCNGDSGGPIFVQHPLYPCLKQVIGIVSYGLVCGSQGLPSVYTKVHLYTDWIESIV
WGN

>CG2045
FSFRLVGGHNTGLFEFPWTTLLEYETVSGGKDYACGASFIAQRWLLTAAHCIHTMGRNLTAAILG
EWNRDTDPDCENDLNGVRECAPPHIRVTIDRILPHAQYSELNYRNDIALLRLSRPVNWLQMQNLE
PVCLPPQRGRYANQLAGSAADVSGWGKTESSGSSKIKQKAMLHIQPQDQCQEAFYKDTKITLADS
QMCAGGEIGVDSCSGDSGGPLTVEANTASGNRYVYLAGVVSIGRKHCGTALFSGIYTRVSSYMDW
IESTIRANRI

>CG11668
SQNLLVGGRLTQENEHPYMCALGWPSRTNRWIHEHGSSKRRYTFNCGCAMIAPRFAITAAHCASV
GGESPSVALIGGVELNSGRGQLIEIKRISQHPHFDAETLTNDLAVVKLARRSHMPVACLWNQESL
PERPLTALGYGQTKFAGPHSSNLLQIMLYHLNFQQCQRYLHNYDKLANGLGSGQMCAGDYSGNMD
TCQGDSGGPLLLHQHMRHHRHTIPYVVGITSFGGACASGQPGVYVRIAHYIQWIEQQVWP

>CG16705 (SPE)

FADRIFGGTNTTLWEFPWMVLLQYKKLFSETYTFNCGGALLNSRYVLTAGHCLASRELDKSGAVL
HSVRLGEWDTRTDPDCTTQMNGQRICAPKHIDIEVEKGIIHEMYAPNSVDQRNDIALVRLKRIVS
YTDYVRPICLPTDGLVQNNFVDYGMDVAGWGLTENMQPSAIKLKITVNVWNLTSCQEKYSSFKVK
LDDSQMCAGGQLGVDTCGGDSGGPLMVPISTGGRDVFYIAGVTSYGTKPCGLKGWPGVYTRTGAF
IDWIKQKLEP

>CG32260
TSNRVVGGMEARKGAYPWIAALGYFEENNRNALKFLCGGSLIHSRYVITSAHCINPMLTLVRLGA
HDLSQPAESGAMDLRIRRTVVHEHFDLNSISNDIALIELNVVGALPGNISPICLPEAAKFMQQDF
VGMNPFVAGWGAVKHQGVTSQVLRDAQVPIVSRHSCEQSYKSIFQFVQFSDKVLCAGSSSVDACQ
GDSGGPLMMPQLEGNVYRFYLLGLVSFGYECARPNFPGVYTRVASYVPWIKKHIASA

>CG11313
AYNQITKGNETVLTEFAWMVLLEYRPHDGQQLRTYCAGSLINNRYVVTAAHCVSAATRARKGDVS
FRVSVRLGEHNTSAVVDCLNGRCLPEPVQIAVEEIRIHESFGTRLFWNDIALIRLAREVAYSPSI
RPVCLPSTVGLQNWQSGQAFTVAGWGRTLTSESSPVKMKLRVTYVEPGLCRRKYASIVVLGDSHL
CAEGRSRGDSCDGDSGGPLMAFHEGVWVLGGIVSFGLNCGSRFWPAVYTNVLSYETWITQNIRP

>CG16710
PAYRIFGGEETQPNELPWMALILYAHRSRSVWNERLVSRCAGSLITNRYVLTAAHCLRITGLDLR
RVRLGEHNILSNPDCVTHINGREHCAPEHLEIDVDLSIKHRHYMVFEERPYNDIALLRLKFPVRN
HKLQIAGWGLSHKQGYSNVLLQAYVNGRNADECSLSEPSLGLDKETHICAGNLGGNDTCKGDSGG
PLMAIMERGDEEFVYLAGITSYGYSQCGYGPAAYTKTSKFVEWILWNMYTNIFQ

>CG7432
STGRIVGGVEAPNGQWPWMAAIFLHGPKRTEFWCGGSLIGTKYILTAAHCTRDSRQKPFAARQFT
VRLGDIDLSTDAEPSDPVTFAVKEVRTHERFSRIGFYNDIAILVLDKPVRKSKYVIPVCLPKGIR
MPPKERLPGRRATVVGWGTTYYGGKESTSQRQAELPIWRNEDCDRSYFQPINENFICAGYSDGGV
DACQGDSGGPLMMRYDSHWVQLGVVSFGNKCGEPGYPGVYTRVTEYLDWIRDHTRD

>AAM50955 (CLIP38)
RSNRIVGGHSTGFGSHPWQVALIKSGFLTRKLSCGGALISNRWVITAAHCVASTPNSNMKIRLGE
WDVRGQEERLNHEEYGIERKEVHPHYNPADFVNDVALIRLDRNVVYKQHIIPVCLPPSTTKLTGK
MATVAGWGRTRHGQSTVPSVLQEVDVEVISNDRCQRWFRAAGRREAIHDVFLCAGYKDGGRDSCQ
GDSGGPLTLTMDGRKTLIGLVSWGIGCGREHLPGVYTNIQRFVPWINKVMANDNA

>CG8870
SRRKPTKGKIPALNEFPWMAMLLYGNKNNLSQKLVPKCGGSLINNWYVLTAAHCVEYPFMDYPYA
LKTVRLGEHNTSTNPDRAIVNGRRQYAPLYMEIEVDQIITHEQFNRGRRLINDIALVRLKFPVRY
TRAIQPICLPRAQKLAAHKRKFQASGWPDMGQGIASEVLLRSFIAERHPDVCKSNYDFNLGSQIC
AGGLDGNDTSPGDSGGPLMETVIRGKVTLTYAAGIISYGQKPCVLKTCKPAFYTKTSYFFEWIKS
KLQSPFIDSDSKSRTRK

>CG9737

VTNRIYGGEIAELDEFPWLALLVYNSNDYGCSGALIDDRHILTAAHCVQGEGVRDRQGLKHVRLG
EFNVKTEPDCIEEPNYLSCADAALDIAYEKIHVHPEYKEFSNYKYNDIAIIRLKHPVSFTHFVMP
ICLPNKSEPLTLAEGQMFSVSGWGRTDLFNKYFINIHSPIKLKLRIPYVSNENCTKILEGFGVRL
GPKQICAGGEFAKDTCAGDSGGPLMYFDRQHSRWVAYGVVSYGFTQCGMAGKPAVYTNVAEYTDW
IDSVVQQRKKSQQTQDKMA

>CG13744
LQKRIIGGRPAQFAEYPWQAHIRIAEYQCGGVLISANMVATAAHCIQQAHLADITVYLGELDTQD
LGHIHEPLPVEKHGVLQKIIHPRFNFRMTQPDRYDIALLKLAQPTSFTEHILPICLPQYPIRLIG
RKGLIAGWGKTEAHMGHAGTNMLQVASVPIITTLDCIRWHESKQINVEIKAEMFCAGHSDGHMDA
CLGDSGGPLVIKERGRFVLVGITSAGFGCGVDHQPGIYHNVQKTVRWIQEVVARNEP

>CG3066 (MP2)
FSNKVYNGNDTAIDEFNWMALLEYVDNRGRRELSCGGSLINNRYVLTAAHCVIGAVETEVGHLTT
VRLGEYDTSKDVDCIDDICNQPILQLGIEQATVHPQYDPANKNRIHDIALLRLDRPVVLNEYIQP
VCLPLVSTRMAINTGELLVVSGWGRTTTARKSTIKQRLDLPVNDHDYCARKFATRNIHLISSQLC
VGGEFYRDSCDGDSGGPLMRRGFDQAWYQEGVVSFGNRCGLEGWPGVYTRVADYMDWIVETIRP

>CG5909
GNPKVSGGKTARPGDFPWVALLKYKINDPRPFRCGGSLISERHILTAAHCIIDQPEVIAVRLGEH
DLESEEDCHYLGGTNRVCIPPYEEYGIEQIRVHPNYVHGKISHDVAIIKLDRVVKEKSHIKPVCL
PIDQKSQELDFDQSFFVAGWGGTEKETVATKLQQALITRKSLNECRQYYNKGEVSDNHICATGTG
IKHTCQGDSGGPVFFKHRFKNTYRVVQYGVVSFGGRLCGQNQPGVFASVIDMLPWITQNLQ

>CG9733
IRNRIYDGQDTDVNEFPWMVLLEYRRRSGNGLSTACAGSLINRRYVLTAAHCLTGRIEREVGTLV
SVRLGEHDTRTAVDCPPGGGSCSPEVQRLGFEEIRVHERYSEKASNQVHDIGLIRMERNVRYSDN
IQPICLPSSVGLESRQSGQQFTVAGWGRTLKMARSAVKQKVTVNYVDPAKCRQRFSQIKVNLEPT
QLCAGGQFRKDSCDGDSGGPLMRFRDESWVLEGIVSFGYKCGLKDWPGVYTNVAAYDIWIRQNVR
A

>CG1299
YFKKIVGGEVSRKGAWPWIALLGYDDPSGSPFKCGGTLITARHVLTAAHCIRQDLQFVRLGEHDL
STDTETGHVDINIARYVSHPDYNRRNGRSDMAILYLERNVEFTSKIAPICLPHTANLRQKSYVGY
MPFVAGWGKTMEGGESAQVLNELQIPIYDNKVCVQSYAKEKRYFSADQFDKAVLCAGVLSGGKDT
CQGDSGGPLMLPEPYQGQLRFYLIGVVSYGIGCARPNVPGVYSSTQYFMDWIIQQVQDTP

>MsHP6
LDLHILGGEEASLGEFPHMVALGFDNGGGEYRFDCGGSLISNYYVLTAAHCIDTADREPPSVVRA
GVVNIGGPAWDDETDYRVAETILHPNYTRREKYHDVALLRLDRPVQFSSTLNAVCLFSSNENPTS
KLTITGWGRTSNTRDIKSSKLLKADVVVVPSDKCGESYTNWRKLPHGISQEMMCAGDPKGVRDTC
QGDSGGPLQLMEKDGLYRLVGVTSFGRGCGSYVPGVYTRVSNYLGWIESIVWPN

>MsHP8

NNDRIVGGIQTEIDEHPWMVLLRYDKPSGWGFYCGGVLISSKYVLTAAHCVKGSDLPPNWKLSQV
RLGEWNTSSQVDCVGDDCSQPVQDIRIEQIVAHESYDPEDNNQQNDIALLRLAQNVHLNDFVKPI
CLPTTEDLRDSNFDGLEMEVAGWGKTETRTESDVKLKVRVPVVSRRLCKSVYERVERLITDKQLC
AGGVEGKDSCRGDSGGALMGQAPSANNWLVVGVVSYGPSPCGTPGWPGVYTRVGAFMDWILSKLR
P

>MsHP13
ADSLIVGGKDADRNEFTHMVLLGFGEDPRKVKWDCAGSLISEYFVLTAAHCVTSADSGNVTYASV
GVLTRSEVAPDNTYKISERFRHPSFRRGVYNDIALLRLEREVLLGEYRVPACLHVGDTVKDARAM
ATGWGLLEYRGNVSDILQKVTLKKYRSRICKAIFPPHTLVSHNYDEKTQLCFGGYNDTQPGDTCN
GDSGGPLMIKHKKINCMYLILGVTSGGRGCAWRGKPGLYSRVSHYISWIESVVWR

>MsHP15
VGNKIIGGNATAINEYPWLVIIEYEHPIEKTKLMCGGALISGKYVLTAAHCVSGAILNEGTPKFV
RLGEYNITNKGPDCVPSAFGDPDCTDDMILAPIEEIIVHPEYDRFDLDKRHDIALIRLKIYAPYT
DFIRPICLPKVDYSQSPPADLSFYVAGWGRYIENTTAKIYRRSSVKLHVEVPYVVRDQCQAAIRT
IPGVENIVFRSGQICAGGVLGQDSCKGDSGGPLMYENKETRKYEVVGIVGSGAQECGQPGIPGVY
TYIYEYLPWIRQNIRV

>MsHP21
ADDLIIGGQNASRNEFPHMALLGYGEEPDVQWLCGGTLISENFILTAGHCISSRDINLTYVYLGA
LARSEVTDPSKQYRIKKIHKHPEFAPPVRYNDIALVELERNVPLDEWLKPACLHMGDETADDRVW
ATGWGLTEYKASSGANILQKVVLNKFSTFECILQYPPHRLMSQGFDVNSQMCYGDRSQSKDTCQG
DSGGPLQIKHKKINCMWLIIGVTSFGKACGFIGEPGIYTKVSHYIPWIESVVWP

>MsPAP1
NGDRIYGGQITDLDEFPWMALLGYLTRTGSTTYQCGGVLINQRYVLTAAHCTIGAVEREVGKLIT
VRLGEYDTQNSVDCVDDVCADPPQNIPIEVAYPHSGYSDNNKNRKDDIALVRLTRRAQYTYYVKP
ICLANNNERLATGNDVFVAGWGKTLSGKSSPIKLKLGMPIFDKSDCASKYRNLGAELTDKQICAG
GVFAKDTCRGDSGGPLMQRRPEGIWEVVGIVSFGNRCGLDGWPGVYSSVAGYSDWILSTLRSTNV

>MsPAP2
FDNKILGGEATAIDQYPWLALIEYHKLAEIKLMCGGSLISAKYVLTAAHCVKGPILEKGTPKNVR
LGEYNTTNNGPDCVPSDAGSQDCTEGMVLAPIEQTIPHPKYKPYSLNKQHDIALIRLRTFAPRTD
FISPICLPKIDYAQSPPSAFSLYVAGWGRYIQDVEAGIYRSSKIKLHVNVPFVDNERCLGGVRKL
RNGENISLWKGQLCAGGVSGKDSCKGDSGGPLMYDKERKYEAVGVVSYGAEICGQQGIPGVYTNV
HEYLPWIKATIKA

>MsPAP3
VGNKIIGGNATDVDQYPWLTIIEYVKTGPIKLLCGGVLISGKYVLTAGHCLTGPVLQIGTPTNVR
LGEYNTKNDGADCVTVEAGGMDCTEGAVIVPIEKTIPHPEYNPISRTRRNDIGLIRLKEMAPFTD
FIRPICLPSLDLTQAPPVNFTLYAAGWGAVSTSQPSSNVKLHVQLPFISYERCQPSYAVQNRQIE
LWEKQVCAGGEAGKDSCKGDSGGPLMYENGQTYEVIGIVSFGPTPCGMQDIPGVYTKVHSYKDWI
ISNIKP

>AgCLIPB8
YVAKIRGGQLAEIDEFPWMAMLLYERDNNALTQGCGGALISRTYVITAAHCVTGKNFQQTKGRLK
FVRLREYNIHTNPDCVYENDLKDCSDDMIDLVPQAVIPHPEYDSESSNQQHDIALIRIEQTPPFT
DFLRSICLPEQNFESSATPGKKLSVSGWGRTDIFKDNLGPDVLSPIKLKLSLPYVEREKCSKTFR
PWSFALGPGQMCAGGERAKDTCAGDSGSPLMSYDMKRAIWYITGIVSLGVRGCGVEGLPGVYTNV
HHYLPWIKMYTGA

>AgCLIPB9
LADRIIGGNYTAIDEFPWYALLEYQSKKGERAFKCGGSLINGRYVLTAAHCLANKKLDEGERLVN
VRLGEYNTATDTDCADGNPDDCADPPQNFGIEAQIVHPGYDKNGPYQHHDIALIRLDRDVTMNNF
VSPVCLPPDDFPPTSPGLNVTAVGFGHTGRQRHSGIKKKAQFPVFAQEECDKKWKNIEVIGEQLC
AGGVFGIDSCSGDSGGPLMVKRFYWIQEGVISFGNQCALEGWPGVYTRVSSYLDWIRQNIRR

>AgCLIPB10
IGMRIYGGQNADIDEFPWLALLQYENRKGERKYSCGGSLINRRYVLTAAHCVIGEVERKEGKLVS
VRLGEYNTKTEIDCVTEEQEEICADPPIDAGIESVIVHPGYQDMAHADDIALLRLAQSIEYTSFV
QPVCLPLTDFRASKTGEVNFVTGFGRTLQESRSAVKQKLGIKVYDHARCQEKYATKNSSITTNQL
CAGGEYAKDSCHGDSGGPLMKLQKVWYLEGIVSYGNRCGLEDWPGVYTHVPAYMAWVRSNIKE

>BmBAEEase
NNDRIFGGIQTEIDEHPWMALLRYDKPLGWGFYCGGVLIAPMYVLTAAHCVKGSDLPSSWQLSQV
RLGEWNTSTETDCVEGDCSGPVQDIPVQQIIAHENYDPNDKDQQNDIALLRLSRNAQFNDFVSPI
CLPTSNELRQNEFESDYMEVAGWGKTETRSESDVKLKVRVPIVNREECANVYSNVDRRVTNKQIC
AGGLAGRDSCRGDSGGALMGQSPKANNWYVFGVVSYGPSPCGTEGWPGVYTRVGSFMDWILSKLE
Q

>BmPPAE
VGDKIVGGAPASIDSYPWLVVIEYVRLERTMLLCGGALISGKYVLTAGHCVKGGILDVGTPKTVR
LGEYNTTNPGRDCVSVSAGGTDCTDPLVKIGIEKTIPHPDYQPYHFLRKHDIGLIRLQSIAPFTD
FIRPICLPSTDYTVNPPSKFALTVAGWGRYLQFDNGTVRSSKIKLHVTLPFVQRDVCEANQKPLR
NGQRITLWKGQMCAGGEAGKDSCKGDSGGPLMYEHSKKYEAVGIVSFGPEKCGQIDIPGVYTNVY
EYLPWIQNTIEP

>TmSPE
TQNRIYGGEKTDLDEFPWMALVEYEKPGGSRGFYCGGVLISKRYVLTAAHCVKGKDLPKTWKLVSV
RLGEYNTETDTDCINNGFGEDCAPPPVNVQVEARIAHESYEPNNINQYHDIALLRLRREVKFSDY
IKPICLPTTTEELSKSYLGQKLFVAGWGKTENRSESNIKLKVQVPVKQMSDCTATYSSANVRLGS
GQLCAGGESGKDSCRGDSGGPLMILSLDKDKDIHWYAAGVVSFGPSPCGMANWPGVYTKVSKYVD
WIVGKLKP

>TmSAE
IIKLIVGGTNATRKEFPHMAVIGFEPQPGDIKWLCGGTVLSKHYILTAAHCLSHQEHGRARYVRI
GVTDLEDTNHRQQLEVEELIPYPEYKSSSHYHDIGLLRLKRSAKLDSFTVPACLYRKHDIEAEKA

IATGWGHTTWGGSGSNNLLKVTLDLFDHASCNRSYKNQISRRLKDGIIDDIQVCAGSLDDEKDTC
QGDSGGPLQIFHESKDIKCMYDIIGVTSFGKACSGSPGVYVRVSQYIGWIEDIVWPENS

>HdPPAF1

EADKILNGDDTVPEEFPWTAMIGYKNSSNFEQFACGGSLINNRYIVTAAHCVAGRVLRVVGALNK
VRLGEWNTATDPDCYGAVRVCVPDKPIDLGIEETIQHPDYVDGSKDRYHDIALIRLNRQVEFTNY
IRPVCLPQPNEEVQVGQRLTVVGWGRTETGQYSTIKQKLAVPVVHAEQCAKTFGAAGVRVRSSQL
CAGGEKAKDSCGGDSGGPLLAERANQQFFLEGLVSFGATCGTEGWPGIYTKVGKYRDWIEGNIRP
